# Predicting alfalfa leaf area index by non-linear models and deep learning models

**DOI:** 10.3389/fpls.2024.1458337

**Published:** 2024-11-11

**Authors:** Songtao Yang, Yongqi Ge, Jing Wang, Rui Liu, Li Fu

**Affiliations:** ^1^ College of Information Engineering, Ningxia University, Yinchuan, China; ^2^ Ningxia Key Laboratory of Artificial Intelligence and Information Security for Channeling Computing Resources from the East to the West, Yinchuan, China; ^3^ College of Resources Environment and Life Sciences, Ningxia Normal University, Guyuan, China

**Keywords:** alfalfa, leaf area index, non-liner model, deep learning model, MOSUM.

## Abstract

Leaf area index (LAI) of alfalfa is a crucial indicator of its growth status and a predictor of yield. The LAI of alfalfa is influenced by environmental factors, and the limitations of non-linear models in integrating these factors affect the accuracy of LAI predictions. This study explores the potential of classical non-linear models and deep learning for predicting alfalfa LAI. Initially, Logistic, Gompertz, and Richards models were developed based on growth days to assess the applicability of nonlinear models for LAI prediction of alfalfa. In contrast, this study combines environmental factors such as temperature and soil moisture, and proposes a time series prediction model based on mutation point detection method and encoder-attention-decoder BiLSTM network (TMEAD-BiLSTM). The model’s performance was analyzed and evaluated against LAI data from different years and cuts. The results indicate that the TMEAD-BiLSTM model achieved the highest prediction accuracy (R² > 0.99), while the non-linear models exhibited lower accuracy (R² > 0.78). The TMEAD-BiLSTM model overcomes the limitations of nonlinear models in integrating environmental factors, enabling rapid and accurate predictions of alfalfa LAI, which can provide valuable references for alfalfa growth monitoring and the establishment of field management practices.

## 1 Introduction

Alfalfa (*Medicago sativa* L.) is regarded as the “king of forages” upon account of its remarkable forage properties, prolific production, high protein and nutritious content, and good palatability (Baral et al., 2022). Between 2010 and 2020, China’s import of alfalfa hay increased from 200,000 tons to 1.4 million tons in response to the growing market demand (Wang and Zhang, 2023). To reduce import dependence, China actively expanded its alfalfa cultivation area and increased alfalfa production (Wang et al., 2023). However, alfalfa production faces significant challenges due to regional climate variations, limited water resources, and soil conditions (Qian et al., 2020). The leaf area index (LAI) of alfalfa is a crucial indicator for defining the canopy structure of alfalfa, statistically describing the growth and density changes of alfalfa leaf populations (Tooley et al., 2024). It can be used to assess the dynamics of leaf growth and yield in alfalfa (Tripathi et al., 2018). Therefore, predicting the alfalfa leaf area index is significant for monitoring growth dynamics and guiding field management.

Methods for predicting LAI through direct measurement of leaf area are often destructive and can be costly. Numerous studies have developed nonlinear models for LAI prediction by analyzing the optimal growth requirements at each developmental stage of plants. Examples include the Logistic model, Gompertz model, Richards model, and Schnute model (Zhang et al., 2021; Duran and Ünal, 2024). Guo et al. (2022) established a generalized Logistic model using growing degree days (GDD) and relative growing degree days (RGDD) as key parameters to describe the height, leaf area index, and biomass accumulation of maize. Karadavut et al. (2010) was carried out on five maize varieties (Monton, Ranchero, Progen 1550, 35 P12, and TTM 81-19) to explain the fitting performance of the Richards model on leaf data. Chaturvedi et al. (2017) developed allometric models for estimating the leaf area index (LAI) of Tectona grandis (teak) trees across ten diameter classes in India. Non-linear regression models, especially logistic and Gompertz, effectively explained over 60% of LAI variability. The advantages of such models include their simplicity, ease of calculation, and reasonable interpretability. However, the limitations are also obvious. This is mainly reflected in two aspects: first, the parameter settings of such models need to be verified and calibrated through years of field experiments, and their applicability in different regions is poor (Lin et al., 2023; Brogi et al., 2020); second, such models are usually one-dimensional, lacking the consideration of a large number of environmental influences, and the dynamic change of variables over time is not taken into account (Desai et al., 2020).

In contrast, deep learning is more suitable for handling non-linear prediction problems. Deep learning models can improve their predictive ability by self-learning the relationships between data from multiple dimensions based on historical datasets, including crop growth information, crop environmental information, and field management strategies. Ze-hao et al. (2020) used LSTM to model winter wheat temporal LAI and LAI in different growth periods, and the results showed that the LSTM network has good prediction ability. Long Short-Term Memory (LSTM), while capable of obtaining long-term dependent time series estimates from continuous time series data, uses only prior state knowledge and ignores back propagation of information about current vegetation changes (Sherstinsky, 2020). BiLSTM adds a reverse operation based on LSTM, which is better than LSTM at capturing the relationship between sequential features (Siami-Namini et al., 2019). LSTM shows excellent performance in time-series based prediction results, but performs poorly in dealing with data mutations.

LSTM has strong self-learning and self-adaptive ability, which overcomes the shortcomings of classical non-linear models to a certain extent. However, alfalfa is often at the maximum LAI value during the cutting, which leads to LAI data mutations after cutting. The presence of these mutation points may greatly reduce the predictive performance of LSTM. Researchers have proposed detection strategies for the change point problem, including maximum likelihood, least squares, minimum absolute length, and minimum descriptive length (Anastasiou and Fryzlewicz, 2022; Chen et al., 2022). This paper examines the number and location of mutations in alfalfa leaf area index data using moving sum (MOSUM) method. The MOSUM method not only effectively detects data mutations, but also greatly reduces the computational complexity (Burczaniuk and Jastrzębska, 2024).

To overcome the limitations of nonlinear models in integrating environmental factors and the inefficiency of LSTM in handling abrupt changes in LAI data, this study proposes a TMEAD-BiLSTM method for the rapid and accurate prediction of alfalfa leaf area index. The main contributions of this paper are as follows:

1) We established Logistic, Gompertz, and Richards models for predicting alfalfa leaf area index based on growing degree days, achieving a prediction accuracy (R²) greater than 0.78 for all three models.

2) To address the issue of abrupt data changes after alfalfa harvesting, we proposed a TMEAD-BiLSTM model that combines the MOSUM method with a bidirectional long short-term memory (BiLSTM) encoder-decoder neural network. This model predicts the leaf area index (LAI) of alfalfa by utilizing its annual cycle and different planting strategies. The results demonstrate that this deep learning model achieved the highest prediction accuracy (R² > 0.99).

## 2 Materials and methods

This section introduces the experimental dataset, which comes from the publicly available dataset we previously worked on. Secondly, we describe the non-linear model and TMEAD-BiLSTM model used for predicting alfalfa leaf area index, and display the model training parameters and evaluation indicators. Then the training process and techniques used to optimize model performance were discussed. Finally, we propose evaluation metrics for assessing and comparing the accuracy of the models.

### 2.1 Dataset

The acquisition of alfalfa LAI data requires a significant amount of manpower and resources, and adverse weather conditions may affect the accuracy and feasibility of data collection, further increasing the challenge of obtaining continuous time series data. To the best of our knowledge, only we have publicly released the alfalfa leaf area index dataset (Yang et al., 2024a). The dataset provides growth data of alfalfa under different water and nitrogen treatments, as well as meteorological data for the entire growth period of alfalfa and soil moisture data at different depths (0-10 cm, >10-20 cm, >20-30 cm). The dataset includes field trial data from three years of history (2017-2018, 2022), with a 7-day interval for collecting alfalfa LAI data. During the period from 2019 to 2021, the experimental field was utilized for planting silage maize using a rotation method. The dataset contains a total of 955 leaf area index data. The interval between meteorological and soil moisture data is one day. Integrating multiple features is beneficial for expanding the applicability of estimation and prediction models and improving their accuracy. This paper used non-linear regression models and deep learning models to model the alfalfa leaf area index.

**Figure 1:**
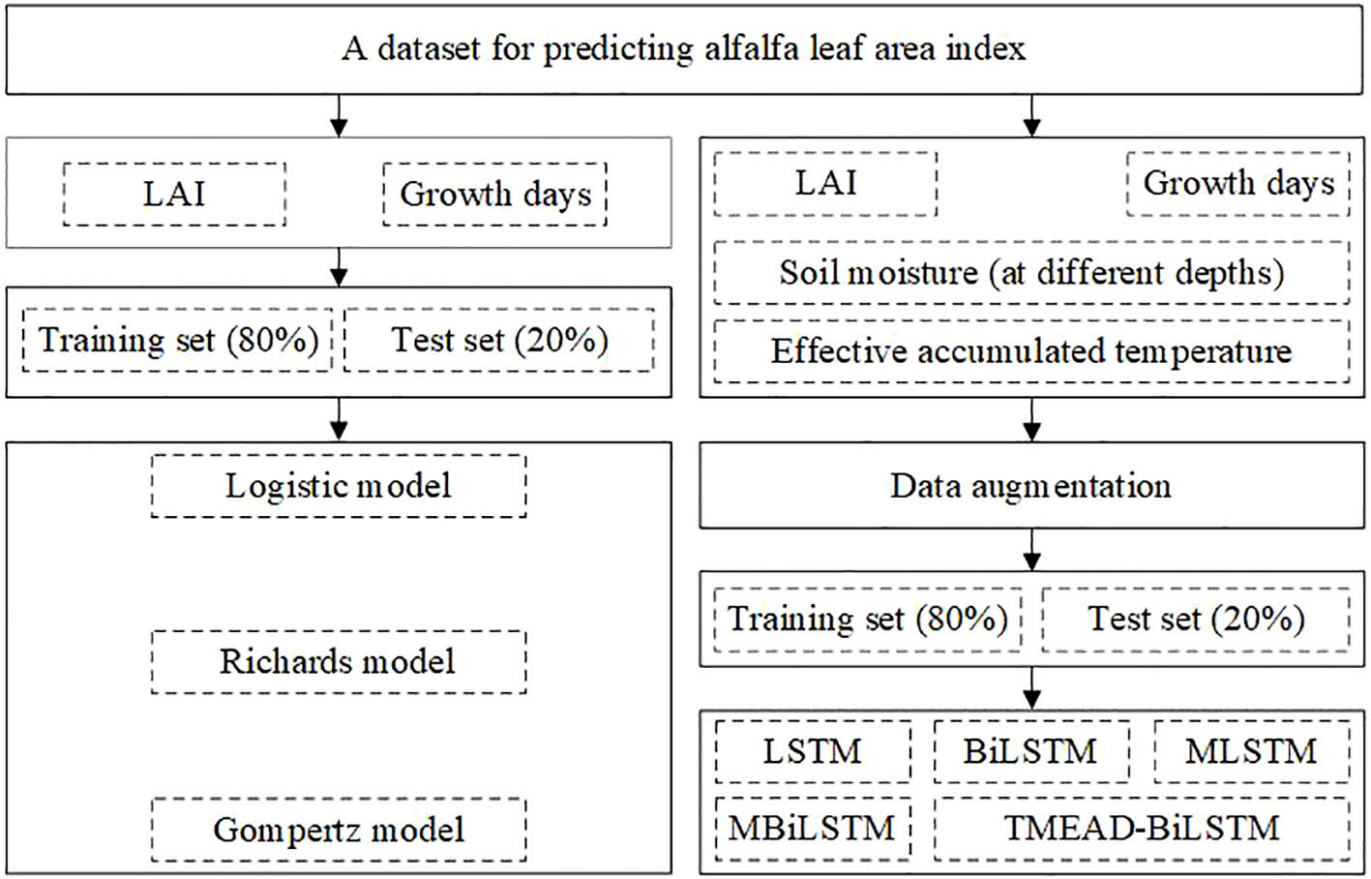
Dataset partitioning and prediction strategies.

We used this dataset to model the alfalfa leaf area index, as shown in **Figure 1**. The dataset contains 955 leaf area index data, the dataset is divided according to the following proportion: (Train: Val = 8: 2): Test = 8: 2. Deep learning models require a large number of samples to participate in training, and we use logistic models with relative leaf area index and growth days for data augmentation. Calculate daily LAI based on the maximum measured LAI value, as shown in equation (14). Thus, a daily LAI dataset with a history of three years is generated, and the partially enhanced dataset is shown in **Table 1**.

**Table 1:** Partial data of LAI dataset.

DateTime	GDD	Growth days	Soil moisture 10	Soil moisture 20	Soil moisture 30	LAI
2018/6/13	194	12	28.45	29.81	25.02	1.26
2018/6/14	212	13	26.88	28.45	24.39	1.40
2018/6/15	231	14	25.62	27.02	23.59	1.56
2018/6/16	245	15	24.65	25.59	22.93	1.74
2018/6/17	260	16	24.71	25.6	22.8	1.95
2018/6/18	278	17	24.15	24.89	22.41	2.18
2018/6/19	297	18	23.23	23.61	21.76	2.43
2018/6/20	318	19	22.15	22.11	21.01	2.71
2018/6/21	337	20	21.14	20.9	20.38	3.01


(1) $$LAI=f(GD)\times LAI_{\max}$$

Where, *f*(**·**) represents the fitting curve; *GD* represents the number of growing days; *LAI_max_* represents the maximum measured LAI value.

### 2.2 Non-liner model

The growth of plants over time typically follows an “S” shaped curve, and such growth characteristics are often simulated using mathematical models such as the Logistic model, Gompertz model, and Richards model. Currently, research on modeling the leaf area index of alfalfa using mathematical models is still in its early stages. In this study, the growth days are taken as influencing factors, and single variable Logistic, Gompertz, and Richards models are used to model four different cuttings of alfalfa. The applicability of Logistic, Richards, and Gompertz models in predicting the leaf area index of alfalfa is evaluated.

While the trend of alfalfa LAI growth days is basically consistent, significant differences in the LAI values among different water and nitrogen treatment plots. The relative leaf area index (RLAI) can eliminate the influence of mathematical models on the fitting of alfalfa LAI under different water and nitrogen treatments, thereby allowing for more accurately analyzing the growth status of alfalfa under different treatments (Su et al., 2022; Liu et al., 2020). Using three non-linear regression models, Logistic (Formula 2), Richards (Formula 3), Gompertz (Formula 4), to describe the trend of LAI changes during plant growth (Nhu et al., 2020; Roosa et al., 2020; Vaghi et al., 2020):


(2) $$RLAI=\frac{LAI}{LAI_{\max}}=\frac{1}{1+e^{a_{1}+b_{1}x+c_{1}x^{2}}}$$

(3) $$RLAI=\frac{LAI}{LAI_{\max}}=\frac{a_{2}}{{\left(1+e^{b_{2}-c_{2}x}\right)}^{\frac{1}{d}}}$$

(4) $$RLAI=\frac{LAI}{LAI_{\max}}=a_{3}e^{-c_{3}e^{-b_{3}x}}$$

Where, *RLAI* is the relative leaf area index; *LAI* is leaf area index; *LAI_max_* is the theoretical maximum LAI. *a_1_*, *b_1_*, *c_1_*, *a_2_*, *b_2_*, *c_2_*, *a_3_*, *b_3_*, *c_3_* and *d* are model fitting parameters; *x* is the growth days.

### 2.3 TMEAD-BiLSTM model

#### 2.3.1 Overall framework

**Figure 2:**
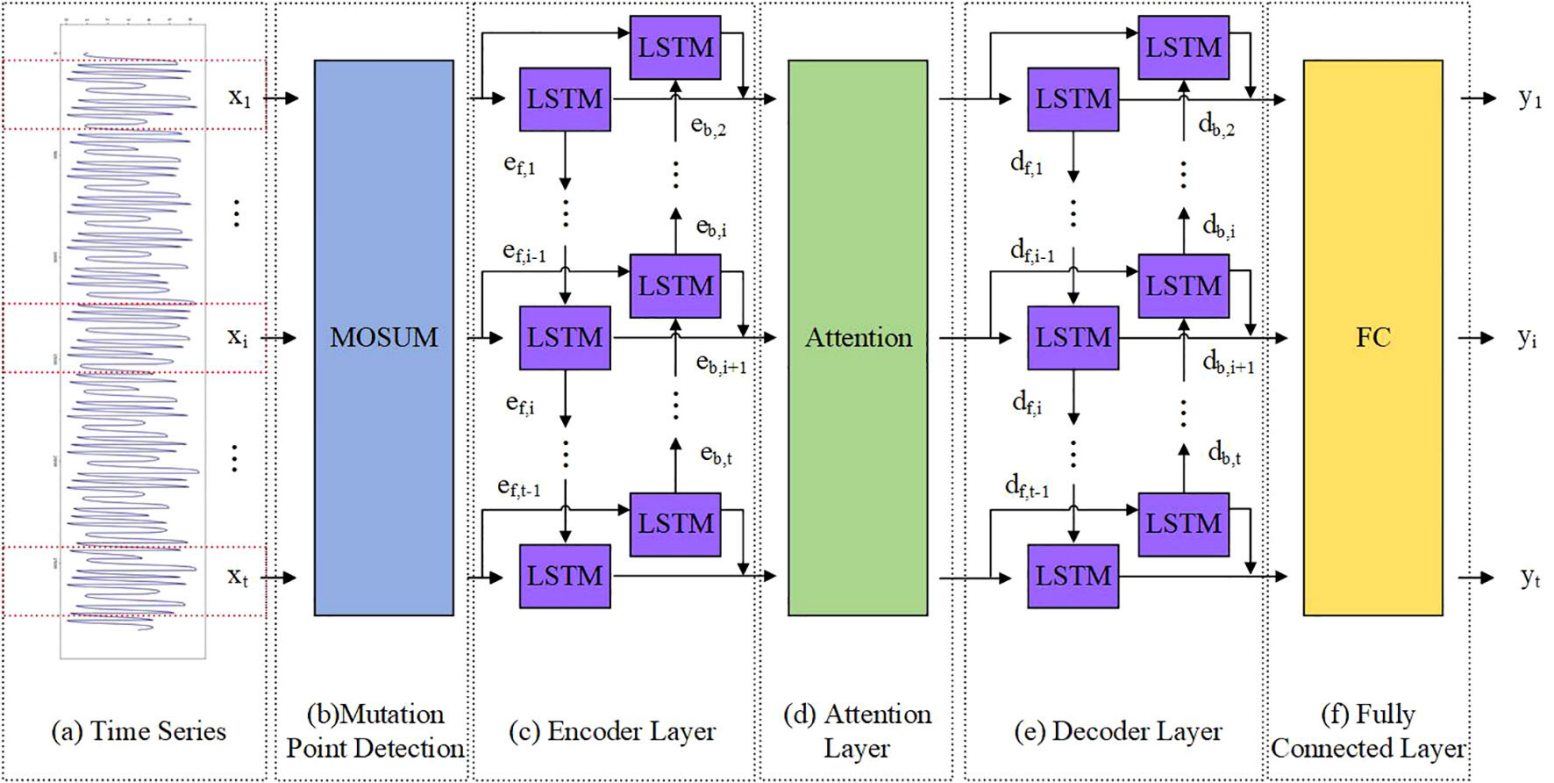
A hybrid model combining attention based BiLSTM encoder-decoder neural network and MOSUM (TMEAD-BiLSTM for short).

The construction of the model can be divided into three parts: (1) The MOSUM method is used to detect mutation points in time series data (**Figures 2A, B**). (2) A model based on encoder decoder was constructed, where both the encoder and decoder are BiLSTM models (**Figures 2C, E**). In order to solve the problem of feature disappearance and information loss caused by covariate feature compression in long sequence data, we introduced an attention mechanism in the model to capture the long-term dependency relationship between input decoder covariates and feature variables (**Figure 2D**). The fully connected layer outputs the predicted results (**Figure 2F**). (3) During the training process, training batches containing mutation points are eliminated through pre-defined functions. This section focuses on the construction process of the TMEAD-BiLSTM model, the overall structure of which is shown in **Figure 2**.

#### 2.3.2 Mutation point detection based on MOSUM

**Figure 3:**
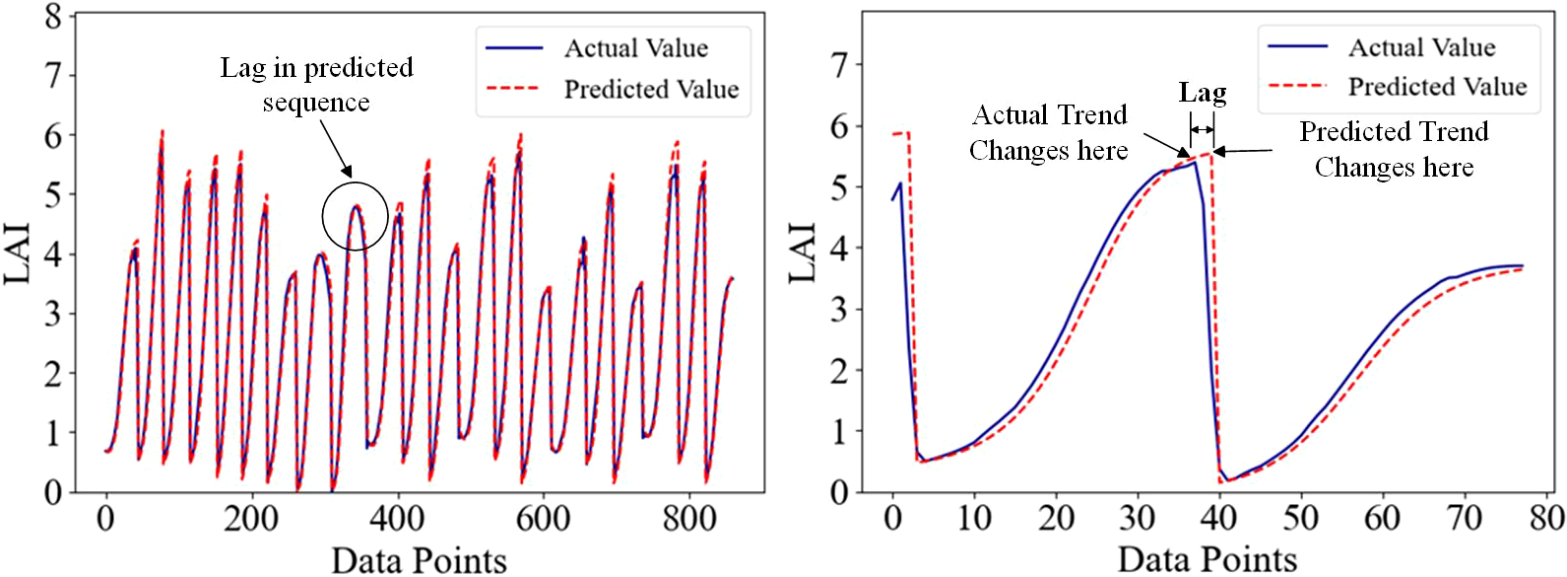
Demonstration of lag in time series forecasting.

Time series data usually contains mutation points, and there is currently no widely recognized method to handle these mutation data points. The currently known processing methods include removing abnormal data or using linear interpolation for filling (Liguori et al., 2021). However, these methods may disrupt the time series properties of the data and affect the accuracy of the analysis. Time series prediction has made remarkable progress in recent years, evolving from traditional statistical methods and machine learning to the latest deep learning techniques, thereby advancing the field of time series analysis (Masini et al., 2023). However, there are still some basic problems in practice, and one of the main problems is the existence of lag difference. Lag differences are deviations or delays that occur in the predicted sequence, which can affect the accuracy and robustness of the model (Samanta et al., 2020). Time series data usually contain abrupt change, and the presence of abrupt change greatly reduces the prediction performance of the model when there is a lag in the prediction process, as shown in **Figure 3**. As a forage crop, alfalfa shows a positive correlation between leaf area index (LAI), and final yield throughout its growth cycle. During mowing, alfalfa LAI was generally reaches its maximum value, and LAI mutations occur afterward, significantly impacting the prediction performance of deep learning models.

The MOSUM method is a technique used to detect structural changes in time series, commonly used for detecting outliers (Eichinger and Kirch, 2018). The core principle is to monitor the evolution of data by calculating the cumulative sum within a sliding window. As the window slides, calculate the magnitude of the cumulative sum change within the current window and set a predetermined threshold. When the cumulative sum changes beyond this threshold, the corresponding window position is marked as an outlier. The MOSUM method utilizes the bandwidth parameter (G value) to adjust the size of the sliding window to adapt to different degrees of variation. The mathematical formula of the MOSUM method is as follows:


(5) $$S_{t}=\sum_{i=t-k+1}^{t}X_{i}$$

(6) $$R_{t}=S_{t}-S_{t-G}$$

Where, *S_t_* represents the cumulative sum of the sliding window in the time series; *R_t_* represents the amount of change in the time series at time *t*; *X_i_* represents the data point in the time series; *k* represents the size of the sliding window; and *G* represents the bandwidth parameter.

**Figure 4:**
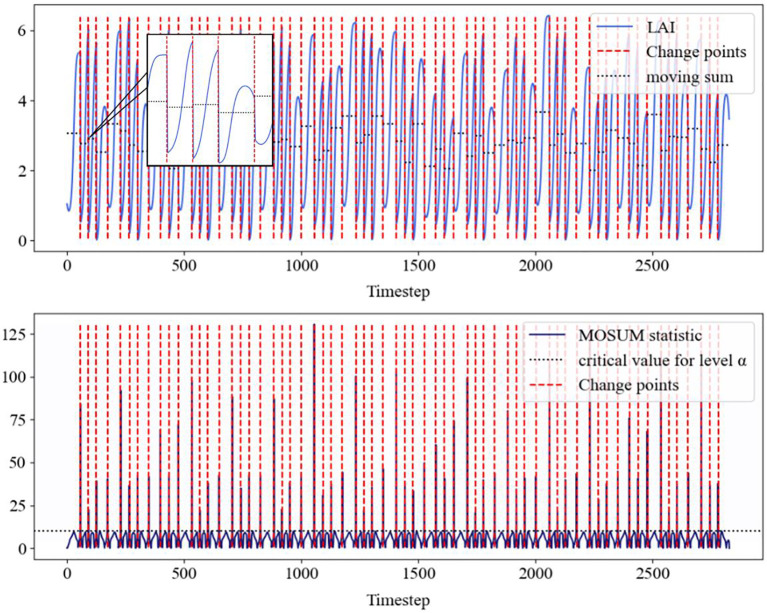
The above figure shows the LAI data of alfalfa detected by MOSUM (blue solid line), the mutation points (red dashed line), as well as the corresponding movement and (black dashed line). The following figure shows the MOSUM statistic, which identifies the mean, threshold (black dashed line), and corresponding change points (red dashed line).

As a forage crop aimed at obtaining plants, alfalfa has a positive correlation between leaf area index (LAI) and crop growth and final yield during its growth cycle. The maximum LAI of alfalfa during the growth period is generally observed during cutting, which leads to LAI mutations and significantly affects the predictive performance of deep learning models. Therefore, we consider using the MOSUM method to detect mutation points in alfalfa LAI data, as shown in **Figure 4**.

#### 2.3.3 Attention with encoder and decoder

The encoder network is a BiLSTM that sequentially transforms the input time series {x_1_,…, x_i_,…, x_t_}. The mapping from *x_i_* to *e_h,i_* and *e_b,i_* can be obtained, as shown in Formula 6–8:


(7) $$e_{h,i}=f_{l}(W_{f}[e_{h,i-l},x_{i}])$$

(8) $$e_{b,i}=f_{2}(W_{\text{b}}[e_{b,i+l},x_{i}])$$

(9) $$encoder\_outputs_{i}=[e_{h,i};e_{b,i}]$$

Where, *e_h,i_* and *e_b,i_* are the forward and backward hidden layer outputs of encoder *i* at time; ***W*** *_f_* and ***W*** *_b_* represent the weight matrices of forward and backward units, respectively; *f_1_* and *f_2_* are units of LSTM; *encoder_outputs_i_* represents the output of the encoder at time step *i*.

Using weighted attention to compute the weighted average of encoder outputs to generate context vectors:


(10) $$attn\_weights_{i}=softmax(e_{h,i}\cdot e_{b,i})$$

(11) $$context=\sum_{i=1}^{T}attn\_Weights_{i}\cdot encoder\_outputs_{i}$$

Where, *attn_weights_i_* represents the attention weights; *context* represents the context vectors.

The decoder network is also a BiLSTM, *d_h,i_* and *d_b,i_* are the hidden layer outputs of the decoder at time *i*. As shown in Formula 11, 12, *d_h,i_* and *d_b,i_* can be obtained:


(12) $$d_{h,i}=f_{3}(W_{f}[d_{h,i-l},x_{i},context])$$

(13) $$d_{b,i}=f_{4}(W_{b}[d_{b,i+1},x_{i},context])$$

Where, ***W*** *_f_* and ***W*** *_b_* indicates the weight matrices of forward and backward units, respectively; *f_3_* and *f_4_* are units of LSTM; *d_h,i_* and *d_b,i_* respectively represent the forward and backward hidden layer outputs of the decoder at time step *i*.

BiLSTM can extract complex features of time series, and attention layer output and hidden state information *d_f,i-1_* 、*d_b,i+1_* can improve the prediction performance. Afterwards, the predicted values are output through the fully connected layer:


(14) $$output_{i}=\text{fc}(d_{h,i},d_{b,i})$$

Where, *output_i_* represents the prediction result; fc denotes the fully connected layer.

#### 2.3.4 Predefined function

**Figure 5:**
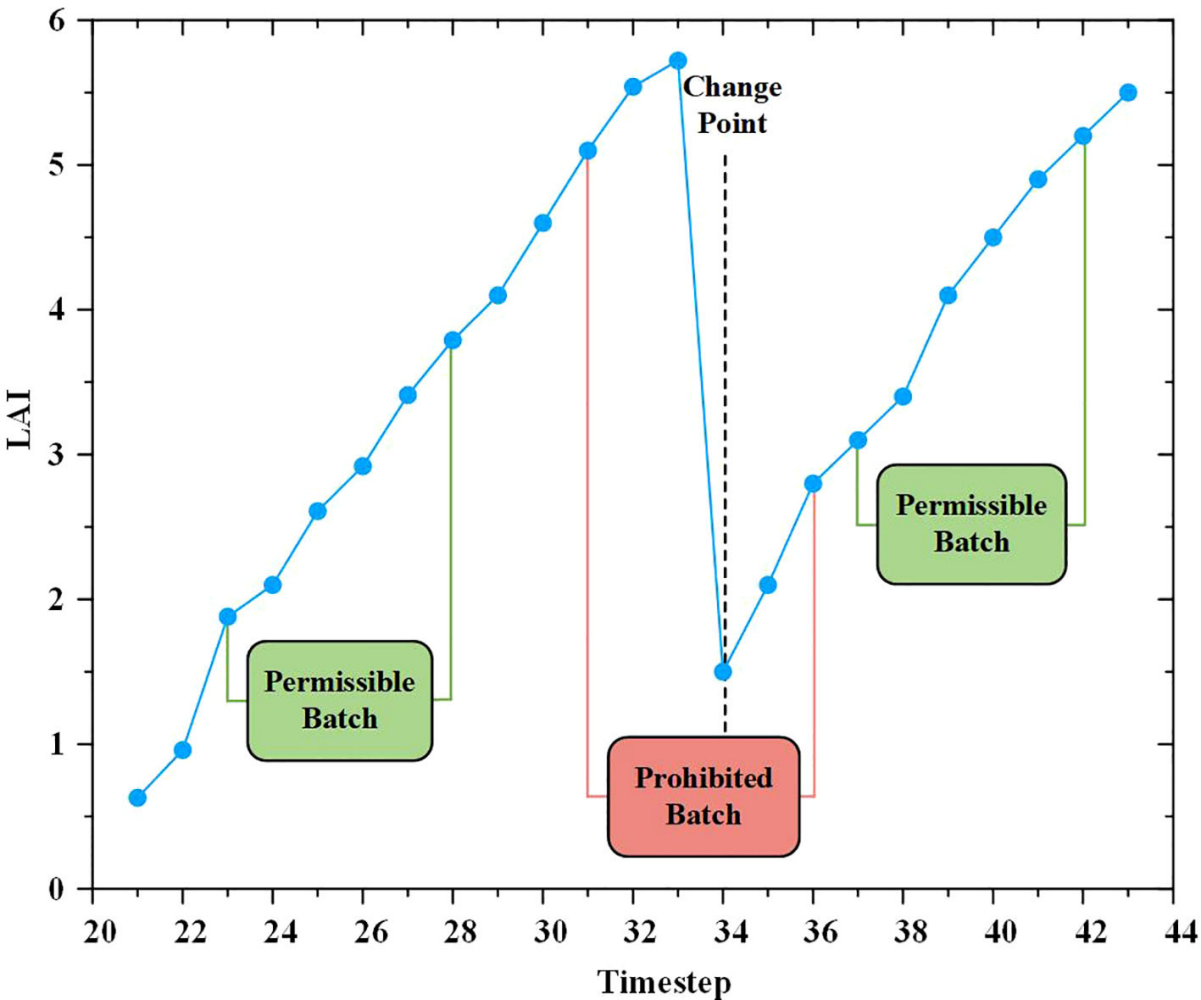
Example of batch processing selection around mutation points. The first green area shows a batch created by choosing t = 23 as the start index, and with a selected batch size of s = 6, the batch contains the points t = 23 to t = 28. The change point is located at t = 34 and is therefore outside the detected batch. The batch is therefore permissible. The red area next to it shows a prohibited batch. The start index of this batch is t = 31 and the end index at t = 36, so the change point is located inside the batch and is not allowed in training. A new batch must be found. The green area to the right indicates another permissible batch, since the change point lies outside of it.

Before the start of training, the MOSUM method is used to detect potential mutation points in the data. When selecting a training batch, predefined functions are used to determine whether to include mutation points in the upcoming training batch. If a mutation point exists, the batch will be skipped until a batch without the mutation point is found. The specific process is shown in **Figure 5**.

In detail, the batch size *s* is one of the most important control parameters of our method. We have to choose *s* in such a way that it could cover at most one change point. If s is larger than the distance between two change points *c_i_*, *c_j_*, the part of the time series between time index *i* and *j* would never be learned. The batches containing this part of the time series would always contain at least one change point and would, therefore, not be allowed. To avoid this, we develop a method that automatically selects the maximum batch size based on the change points. In this method we calculate the distance between all detected change points. We then set *s_max_* to be half of the smallest distance between the change points. Taking half of this distance increases the probability of selecting the allowable batch between the change points, and the resulting number *s_max_* is the maximum batch size that should be selected. See **Algorithm 1** for details.

Algorithm 1 **Find change points and maximum batch size.**

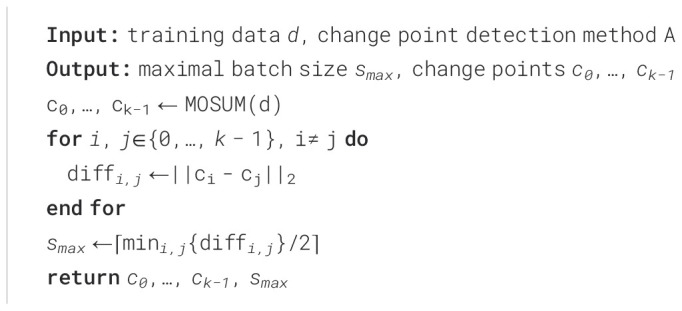

In the TMEAD-BiLSTM model, we pass a batch size *s* ≤ *s_max_* and the detected change points *c_0_*,…, *c_k−1_* to the algorithm. Our goal is to find the start and end points of the batches that do not contain any change points. To do this, we select indices that lie between 0 and n-s-1. For each change point, we check whether the index of the respective change point lies between the previously selected start index and end index of the batch. If the change point lies in the batch, we repeat the method and select a new start index, and perform the same process for this, as shown in **Algorithm 2**. For example, a set of change points has been found through the MOSUM method:

Algorithm 2 **Find valid batch.**

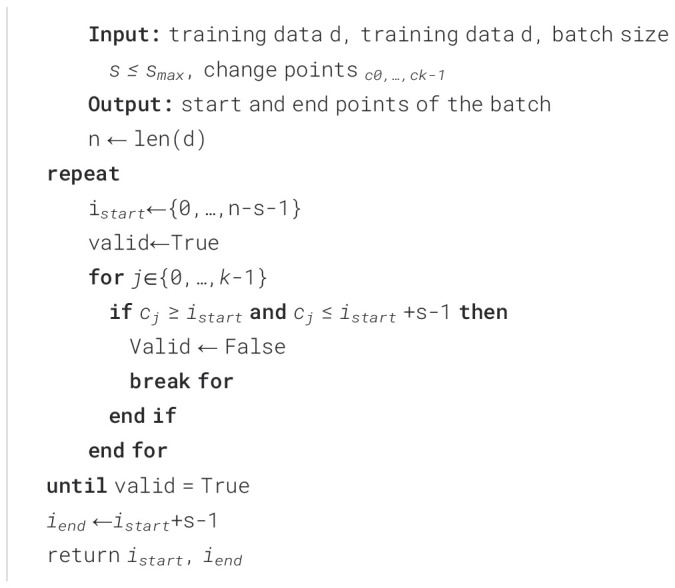

(15) $$C:=\{c_{0},\ldots ,c_{k-1}\}$$

Select batch size *s* ≤ *s_max_* in **Algorithm 1**. Select a random starting index *start*, that is in the range [0, n-s-1] of the time series. This gives us a batch with start point *i_start_* and end point:


(16) $$i_{end}:=i_{start}+s-1$$

Assume that one of the change points lies in the batch, thus without loss of generality ∃j ∈ {0,…, *k*-1} such that *c_j_* ∈[*i_start_*, *i_end_*]. Then again a random start index *i_start’_* is chosen and:


(17) $$i_{end^{\prime }}=i_{start^{\prime }}+s-1$$

If now ∀j∈{0,…, k − 1} holds:


(18) $$c_{j}\notin [i_{start^{\prime }},i_{end^{\prime }}]$$

In the following batch [*i_start’_*,*i_end’_*] is the valid batch.

### 2.4 Model training

Train the hybrid model using the interpolated dataset, with a training-to-testing ratio of 8:2. The encoder and decoder are both BiLSTM with 12 hidden units, using the ReLU activation function. The attention mechanism generates context vectors to enhance output by calculating the correlation between queries and keys. The model uses a learning rate of 0.6e-3 and an Adam optimizer, with a batch size of 32 and a maximum of 300 rounds of training, combined with an early stopping strategy to prevent overfitting. All experiments in this paper were performed on Windows 10 Professional, version 21H2, on a computer with the following main parameters (CPU: AMD Ryzen 5 3600 6-core processor 3.60 GHz; GPU: NVIDIA GeForce GTX 1080 Ti GPU). The code was compiled using PyCharm version 2021.2.3 compiler, and the full syntax followed Python version 3.7.

### 2.5 Evaluation metrics

To prove the prediction performance of TMEAD-BiLSTM and non-linear model, three metrics are adopted to evaluate its prediction accuracy. Specifically, mean absolute error (MAE), coefficient (R^2^) and root mean square (RMSE) are adopted. The R^2^ signifies the agreement between estimated and measured values, with a value closer to 1 indicating a better fit of the model. The RMSE indicates the extent of deviation between estimated and measured values, with smaller values suggesting a better model fit. The MAE assesses the actual deviation between estimated and measured values, with smaller values indicating higher model accuracy. The calculation formula is as follows:


(19) $$\text{MAE}=\frac{1}{\text{n}}\sum_{\text{i}=1}^{\text{n}}|{\hat{\text{y}}}_{\text{i}}-{\text{y}}_{\text{i}}|$$

(20) $$\text{RMSE}=\sqrt{\frac{1}{n}\sum_{i=1}^{n}{\left({\hat{y}}_{i}-y_{i}\right)}^{2}}$$

(21) $$R^{2}=\frac{\sum_{i=1}^{n}{\left({\hat{y}}_{i}-y\right)}^{2}}{\sum_{i=1}^{n}{\left(y_{i}-y\right)}^{2}}$$

## 3 Experiment results

### 3.1 Prediction results annual alfalfa leaf area index

#### 3.1.1 Prediction results of non-linear model

**Figure 6:**
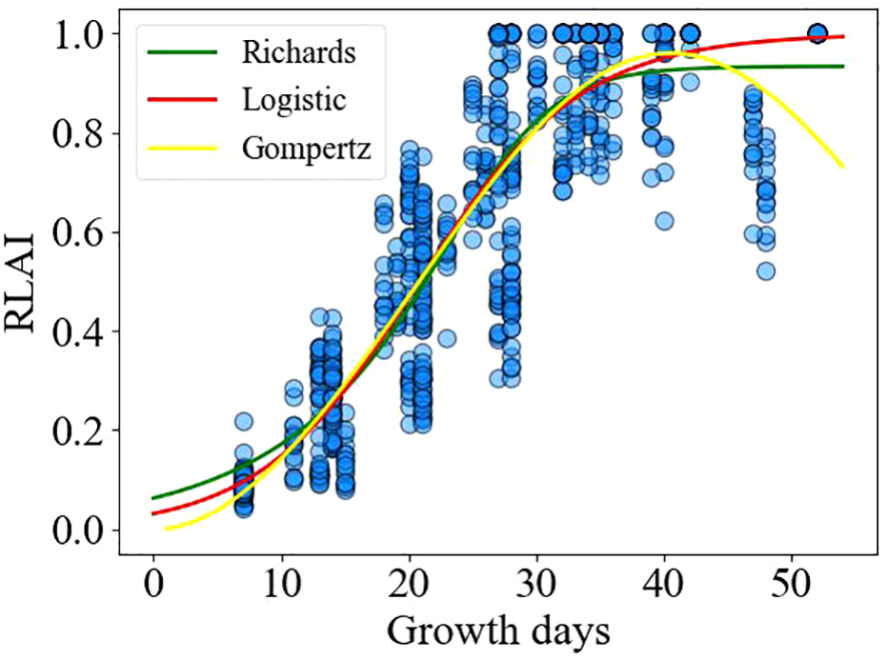
Fitting diagram of annual leaf area index of alfalfa.


The relationship between the relative leaf area index and the number of growing days is illustrated in **Figure 6**. The Logistic, Richards, and Gompertz models were employed to fit the changes in the relative leaf area index over the growing days shown in **Figure 6**, respectively. The fitting results of the logistic (Formula 22), Richards (Formula 23), and Gompertz (Formula 24) models are as follows:

(22) $$RLAI=\frac{LAI}{LAI_{\max}}=\frac{1}{1+e^{2.4249-0.1699x+0.0003x^{2}}}$$

(23) $$RLAI=\frac{LAI}{LAI_{\max}}=\frac{0.9334}{{\left(1+e^{-0.2767-26.7844x}\right)}^{\frac{1}{2.7461}}}$$

(24) $$RLAI=\frac{LAI}{LAI_{\max}}=0.9971e^{-0.1137e^{-6.8693x}}$$

**Table 2** illustrates the fitting and validation results of three non-linear models for the relative leaf area index. It can be observed that all three models exhibit acceptable fits (R^2^>0.78), and the models were validated using test data. It is evident that there is no significant difference in the predictive performance of the three models for the alfalfa leaf area index.

**Table 2:** Modeling and validation results.

Metrics	Fitting results			Verification results		
	Richards	Logisitc	Gompertz	Richards	Logisitic	Gompertz
R^2^	0.7944	0.7892	0.7968	0.8414	0.8362	0.8412
RMSE	0.1421	0.1439	0.1413	0.1191	0.1211	0.1192
MAE	0.1132	0.1112	0.1109	0.0952	0.0936	0.0947

#### 3.1.2 Prediction results of deep learn model

Due to differences in alfalfa phenology under different meteorological conditions and soil moisture, growth days, and soil moisture at different depths (>0-10 cm, >10-20 cm, >20-30 cm) corresponding to LAI at time t were also used as input features. To ensure consistency in input features, the features were normalized and scaled to 0-1. Parallel control experiments were conducted between the TMEAD-BiLSTM model and four models: LSTM, BiLSTM, MLSTM and MBiLSTM. The prediction results of each model are compared using RMSE、MAE and R^2^ measures the degree of fitting of the model. The prediction results of each model are shown in **Table 3**.

**Table 3:** Comparison of prediction accuracy of different LAI prediction models.

Metrics	LSTM	BiLSTM	MLSTM	MBiLSTM	TMEAD-BiLSTM
R^2^	0.8935	0.9179	0.9761	0.9889	0.9986
RMSE	0.5400	0.4857	0.2592	0.1810	0.0662
MAE	0.2857	0.2060	0.2134	0.1406	0.0397

**Figure 7:**
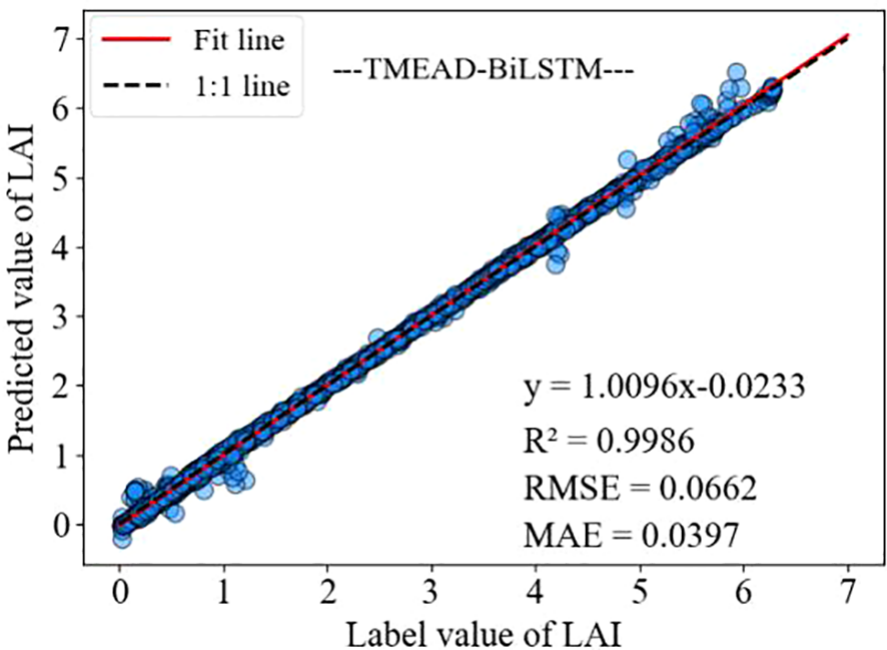
LAI prediction model prediction results.

As shown in **Figure 7**, the model has a high accuracy in predicting 1.5 ≤ LAI ≤ 5.5, with both predicted and labeled values near the 1:1 line. When LAI < 1.5 and LAI > 5.5, the prediction accuracy is slightly lower compared to the previous situation. This may be because under different growth conditions, the time for alfalfa to turn green and set pods is different, and there are significant differences in LAI changes during the corresponding stages of LAI increase and decrease.

The LSTM network performs well in temporal data processing due to its structural features, but the prediction results are not ideal under the influence of multiple feature factors. In contrast, BiLSTM networks have the ability to perceive forward and backward data, enabling stronger causality between forward and backward data. Based on the data presented in **Table 3**, it can be seen that the BiLSTM model has a 27.90% reduction in MAE compared to LSTM. In addition, after adding the MOSUM method, the prediction accuracy of all models was significantly improved, verifying that the MOSUM method can better preserve time series features and effectively reduce the impact of mutation points on model prediction accuracy. To further improve the model’s capacity to mine feature factors and time, this study combines the MOSUM approach with the BiLSTM encoder decoder network model based on attention mechanism. The prediction results show that the R^2^ and RMSE of the TMEAD-BiLSTM model are 0.9986 and 0.0662, respectively.

### 3.2 Prediction results of leaf area index of alfalfa in different cutting

#### 3.2.1 Prediction results of non-linear model

Due to the influence of growth environment temperature, the growth rates of alfalfa vary between different cuttings (Lloveras et al., 1998). It is challenging to simulate the leaf area index changes for multiple cuttings within a year using nonlinear models. Therefore, we modeled the leaf area index of alfalfa for different cuttings separately. Although the variation trend of alfalfa leaf area index with growth days is basically consistent for the same cutting, there are significant differences in LAI values among the experimental plots. In order to analyze its intrinsic mechanism, the relative leaf area index was used to analyze its common growth characteristics.

**Figure 8:**
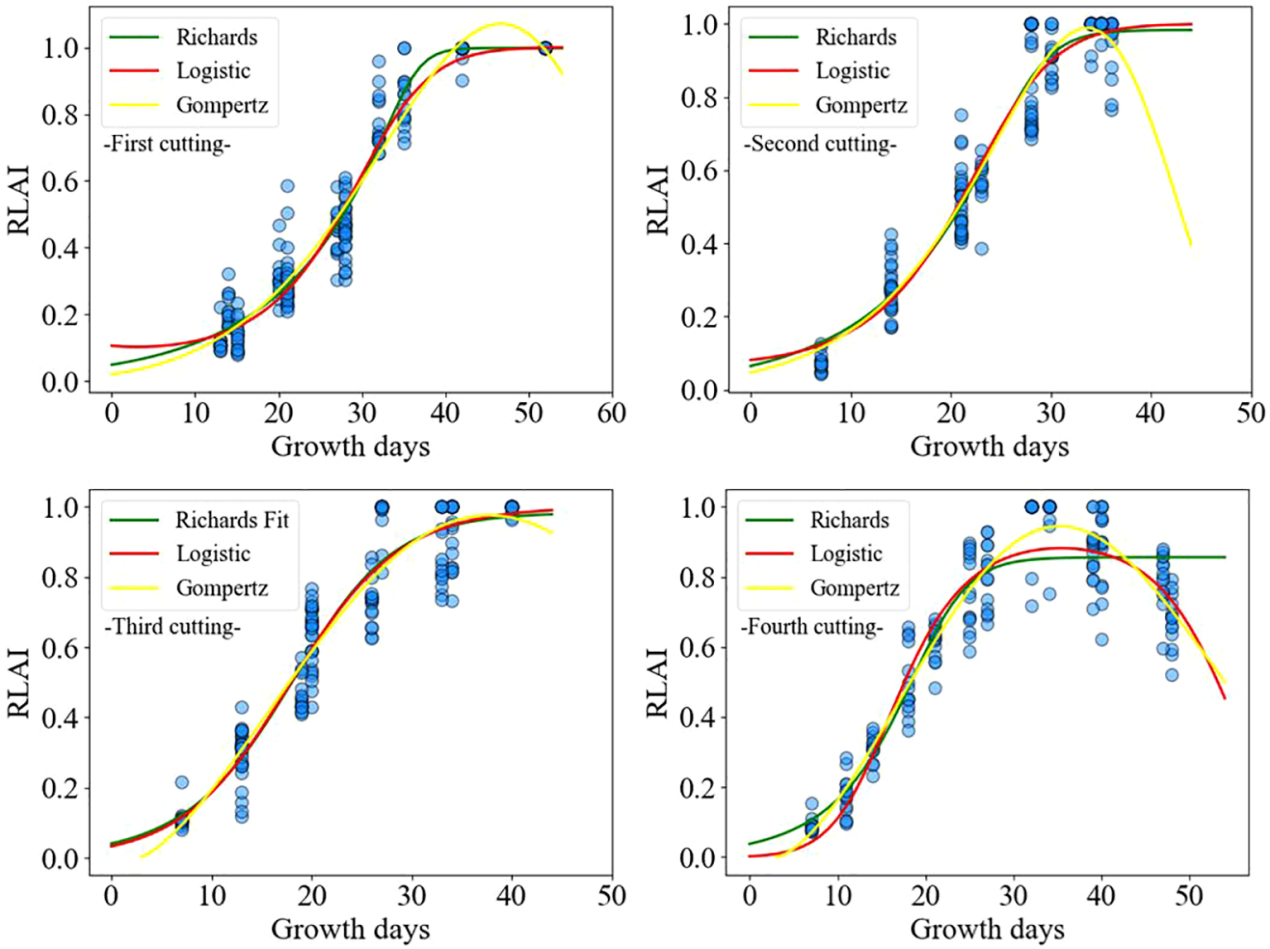
Relationships between relative leaf area index (RLAI) and growth days.

**Figure 8** shows the relationship between the relative leaf area index (RLAI) and growth days. The RLAI of alfalfa was fitted using the Logistic, Richards and Gompertz models. **Tables 4**, **5** presents the fitting effects and fitting coefficients of the models, respectively. The R^2^, RMSE and MAE values of three models were acceptable. Additionally, the maximum LAI of the fourth cutting in the observed region was around 5, and the LAI of the fourth cutting exhibited a declining trend in the later growth period (**Figure 8**). The Logistic and Gompertz models effectively capture the decline process of the leaf area index of the fourth cutting of alfalfa (R^2^ > 0.9). We utilized the Logistic, Richards, and Gompertz models to analyze the rate of change between the alfalfa leaf area index and growth days, computing the first derivative of the fitting curves for different cuttings. Setting growth days to 15, 20, 25, 30, 35, and 40, we averaged the slopes obtained from the three models. The results indicate that the fastest growth rates of alfalfa in different cutting occur at approximately 32, 23, 20, and 18 days after sowing for different cuttings, which is consistent with the results of Yang et al. (2024a)’s study.

**Table 4:** Modeling results for different cutting.

Cutting time	Metrics	Richards	Logistic	Gompertz
First cutting	R^2^	0.9451	0.9403	0.9424
	RMSE	0.0756	0.0800	0.0774
	MAE	0.0507	0.0576	0.0523
Second cutting	R^2^	0.9331	0.9305	0.9353
	RMSE	0.0815	0.0831	0.0802
	MAE	0.0632	0.0649	0.0614
Third cutting	R^2^	0.9091	0.9089	0.9051
	RMSE	0.0902	0.0903	0.0922
	MAE	0.0733	0.0723	0.0722
Fourth cutting	R^2^	0.8698	0.9042	0.9210
	RMSE	0.1057	0.0907	0.0823
	MAE	0.0842	0.0765	0.0650

**Table 5:** Change of leaf area index with growing days.

Non-liner model	Cutting time	a	b	c	d
Logistic	First cutting	2.13	0.02	-0.004	—
	Second cutting	2.42	-0.04	-0.004	—
	Third cutting	3.38	-0.19	0.001	—
	Fourth cutting	5.94	-0.45	0.01	—
Richards	First cutting	0.99	0.64	36.11	7.74
	Second cutting	0.98	0.44	27.78	4.48
	Third cutting	0.98	0.20	19.45	1.25
	Fourth cutting	0.86	0.36	21.17	2.44
Gompertz	First cutting	1.1653	12.9047	0.1768	—
	Second cutting	0.9986	11.6963	0.1919	—
	Third cutting	0.9889	12.3895	0.1152	—
	Fourth cutting	0.9756	12.4646	0.1807	—

The model obtained was validated using the test dataset, and the validation results are shown in **Table 6**. The simulated values of the leaf area index (LAI) based on the Logistic and Gompertz models for alfalfa exhibit a high degree of agreement with the measured values. The R^2^ values for the simulated curves of different cutting times are all greater than 0.9, indicating strong goodness-of-fit. Compared to the Logistic and Gompertz models, the Richards model shows no significant advantage in fitting the data from the first three cuttings, and it performs poorly in fitting the data from the fourth cutting. In summary, the fitting of the Logistic and Gompertz models is satisfactory.

**Table 6:** Verification results of different cutting.

Cutting time	Metrics	Richards	Logistic	Gompertz
First cutting	R^2^	0.9571	0.9546	0.9550
	RMSE	0.0676	0.0696	0.0693
	MAE	0.0473	0.0522	0.0474
Second cutting	R^2^	0.9360	0.9371	0.9371
	RMSE	0.0770	0.0763	0.0763
	MAE	0.0593	0.0593	0.0575
Third cutting	R^2^	0.9244	0.9245	0.9205
	RMSE	0.0766	0.0765	0.0785
	MAE	0.0662	0.0647	0.0656
Fourth cutting	R^2^	0.8582	0.9206	0.9375
	RMSE	0.1044	0.0782	0.0693
	MAE	0.0829	0.0671	0.0552

#### 3.2.2 Prediction results of deep learn model

**Figure 9:**
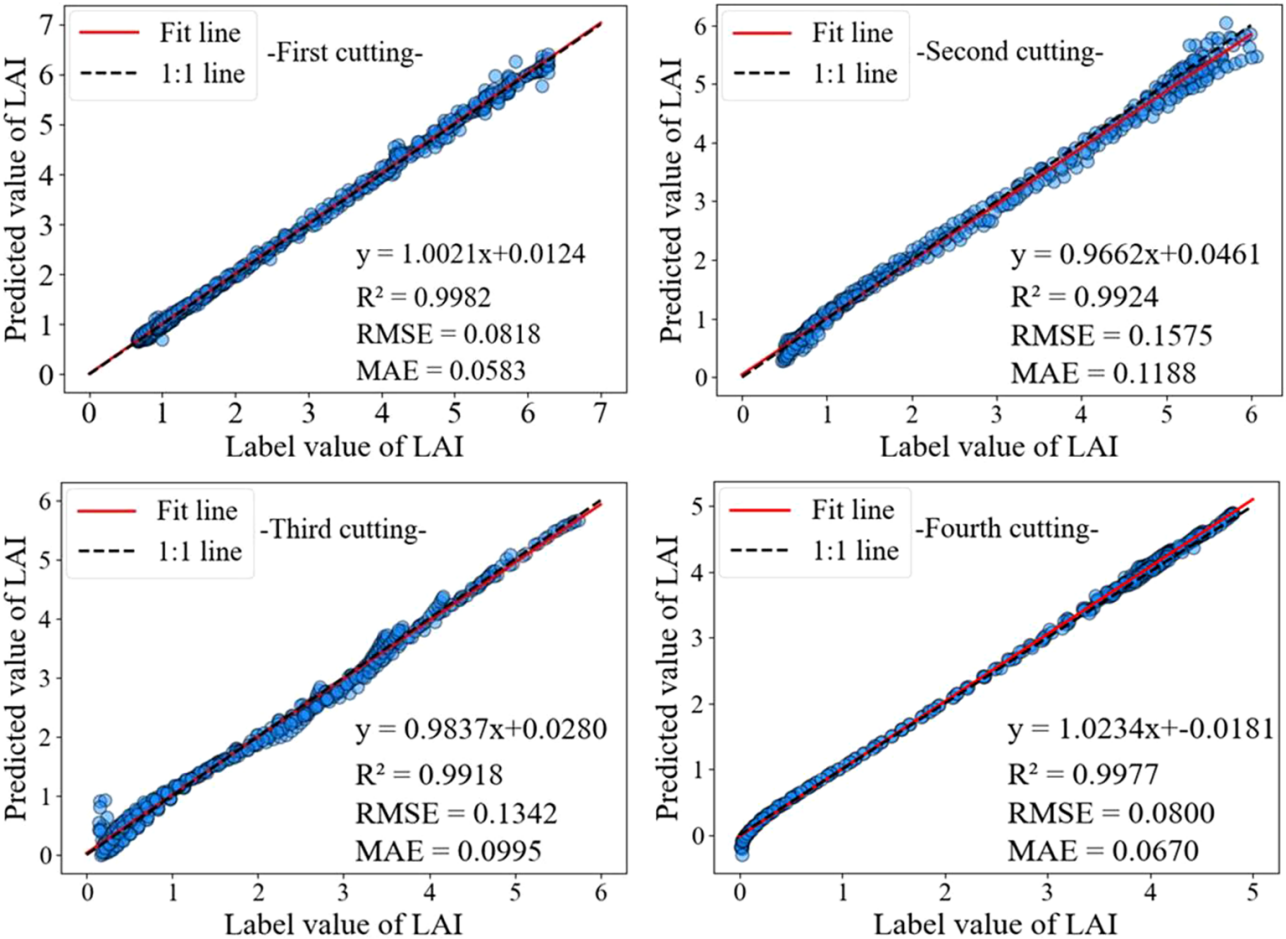
LAI prediction results in different crop cycles.

Alfalfa is harvested multiple times annually, and the publicly published dataset used in this study includes data from 3-4 harvests per year. During the growth process of alfalfa, there are differences in the changes in LAI of alfalfa in different cutting. Therefore, there are differences in the accuracy of LAI prediction models for alfalfa in different cutting. As shown in **Figure 9**, the alfalfa LAI dataset was divided into four batches, namely the first, second, third, and fourth batches. The accuracy analysis of these four batches was performed using the TMEAD-BiLSTM prediction model.

As shown in **Table 7**, the prediction accuracy of the TMEAD-BiLSTM model was higher than that of other experimental models in four different crop cycles. Due to the significant impact of temperature on the growth of alfalfa, the growth periods of the second and third batches of alfalfa are from May to September each year, and the LAI growth rate is relatively high. The model’s ability to capture rapid changes in LAI is insufficient, resulting in a slight decrease in prediction accuracy.

**Table 7:** Comparison of the precision of four LAI prediction results in different cutting.

Cutting time	Metrics	LSTM	BiLSTM	MLSTM	MBiLSTM	TMEAD-BiLSTM
First cutting	R^2^	0.8733	0.9473	0.9857	0.9925	0.9982
	RMSE	0.6287	0.4295	0.2189	0.1638	0.0818
	MAE	0.3307	0.1864	0.1716	0.1235	0.0583
Second cutting	R^2^	0.8342	0.8804	0.9552	0.9766	0.9924
	RMSE	0.6809	0.6173	0.3622	0.2678	0.1575
	MAE	0.3081	0.2699	0.2602	0.2080	0.1188
Third cutting	R^2^	0.8409	0.8916	0.9633	0.9786	0.9918
	RMSE	0.5549	0.4724	0.2728	0.2127	0.1342
	MAE	0.2706	0.2249	0.2260	0.1723	0.0995
Fourth cutting	R^2^	0.8843	0.9508	0.9818	0.9878	0.9977
	RMSE	0.4948	0.3566	0.2008	0.1726	0.0800
	MAE	0.2063	0.1837	0.1710	0.1458	0.0670

## 4 Discussion

### 4.1 Comparative analysis of model accuracy

In this study, non-linear and deep learning models were used to predict alfalfa LAI. The results indicate that the prediction accuracy of the TMEAD-BiLSTM model is the highest, followed by the baseline time series forecasting models (LSTM, BiLSTM, MLSTM, MBiLSTM), while the logistic, Richards, and Gompertz models exhibit the poorest predictive performance (R^2^ > 0.78), with no significant difference observed among the three nonlinear models in terms of predictive efficacy. In contrast to traditional nonlinear models, deep learning models have the capability to integrate multiple environmental factors (e.g., water and nitrogen treatments, soil moisture, meteorological data) for LAI prediction (Cheng et al., 2022). This may account for the superiority of deep learning models over other methods. Extensive studies have revealed that the growth of alfalfa is influenced by various environmental factors (Kaiwen et al., 2020; Liu et al., 2021). Based on our previous work on the dataset, we found that even under the same growth days, the leaf area index (LAI) of alfalfa varies under different water and nitrogen treatments. This observation is consistent with the findings of Ma et al. (2024) and Feng et al. (2016). The dataset we provide includes LAI data from the years 2017-2018 and 2022. The year 2022 marks the first year of alfalfa planting, and the planting period experienced higher temperatures in the region. Alfalfa growth is highly sensitive to temperature, leading to substantial variability in LAI across different growing years. This observation aligns with the studies by Ren et al. (2021) and Kim et al. (2023). These factors also contribute to the decreased accuracy in fitting non-linear models.

Different alfalfa cuttings exhibit significantly different growth rates, thus the use of nonlinear models significantly improves the accuracy of fitting the leaf area index (LAI) for different cuttings. The LAI of the fourth cutting shows a declining trend in the later stages of growth, consistent with the findings of Bai and Bao (2002) The Logistic and Gompertz models effectively captures this changing trend. We observe that the baseline time-series models (LSTM, BiLSTM) have the poorest predictive accuracy. This is attributed to the lagged effect in predictions and the presence of breakpoints in the LAI time-series data, consistent with the findings of Samanta et al. (2020). We use the MOSUM method to achieve mutation point detection and eliminate training batches containing mutation points through predefined functions, avoiding damage to the original dataset and the impact of data mutations on prediction accuracy. Despite the predictive accuracy of three nonlinear models for alfalfa LAI across different cuttings has significantly improved, the differences in growth environments across different years still constrain the predictive performance of nonlinear models, especially concerning temperature and soil moisture, consistent with the findings of Yang et al. (2024b) and Su et al. (2015).

### 4.2 Analysis of Model Complexity and Applicability

In the prediction of alfalfa leaf area index (LAI), deep learning models and nonlinear models each have their advantages and disadvantages. Deep learning models can handle multiple features, including effective accumulated temperature, growth days, meteorological data, soil moisture, and leaf area index, making them suitable for complex environments. However, they have high computational complexity *O*(*T*×*n*×*m^2^*) and space complexity *O*(*n*×*m+m^2^*), with a large number of parameters (for instance, BiLSTM can reach 8×(*m*×*d+m^2^*)), leading to significant memory usage (Shah and Bhavsar, 2022; Maji and Mullins, 2018). Here, *n* represents the sequence length (i.e., number of time steps), *m* represents the number of hidden units, *T* represents the number of training iterations, and *d* represents the dimensionality of the input features (such as the total number of effective accumulated temperature, growth days, meteorological data, soil moisture, and leaf area index). In contrast, nonlinear models rely only on growth days and leaf area index, resulting in low complexity (time complexity *O*(*T*) and space complexity *O*(*n*), with fewer parameters (usually 3-5), and high computational efficiency (De Cock et al., 2017). For example, when processing thousands of samples, the training time for the Logistic model is only a few seconds, and the memory usage is less than one-tenth of that of deep learning models (Banerjee, 2007).

Deep learning models typically exhibit superior suitability for supporting offline predictions compared to nonlinear models (Jin et al., 2022). This preference stems from the inherent characteristics of deep learning architectures, which can leverage pre-trained weight parameters for offline inference without necessitating real-time computations or extensive computational resources. Once the training phase is completed, deep learning models can be seamlessly deployed on relatively modest hardware setups for prediction tasks, rendering them particularly advantageous in scenarios characterized by resource constraints or where continuous online computation is impractical. Conversely, nonlinear models often entail recalculating parameters with each prediction, making them better suited for online prediction scenarios or applications requiring real-time computations (Kalhor et al., 2010). This distinction underscores the versatility and efficiency of deep learning models in facilitating offline predictions, thereby catering to a diverse array of research and practical applications within various scientific domains. Deep learning models can integrate multiple environmental factors for LAI prediction, including water and nitrogen treatment, soil moisture, and meteorological data. This ability significantly improves prediction accuracy, but also increases computational load and parameter complexity (Justus et al., 2018). In addition, deep learning models are suitable for predicting leaf area index (LAI) in different ecological environments due to their powerful feature extraction ability and the advantage of cross regional prediction through transfer learning. They can quickly deploy data from the source region in new areas and achieve good prediction results (Zeng et al., 2020). Although non-linear models perform well in specific regions, their applicability is mainly limited to local data due to the lack of generalization ability and adaptability to cross regional data, making it difficult to effectively meet the needs of cross regional prediction. However, the training of deep learning models requires the participation of large datasets to effectively capture complex patterns and interactions in the data (Najafabadi et al., 2015). The implementation of deep learning models may be challenging in resource constrained environments or with limited data availability (Liu et al., 2024). In this case, traditional nonlinear models provide a practical solution that balances the trade-off between computational efficiency and predictive performance.

## 5 Conclusion

To overcome the limitations of non-linear models in incorporating environmental factors and the inefficiency of LSTM in handling abrupt changes in LAI data, we proposed a TMEAD-BiLSTM model. This model was compared with classical non-linear models such as Logistic, Gompertz, and Richards. The TMEAD-BiLSTM model demonstrated superior predictive accuracy compared to the non-linear models. The strength of classic non-linear models lies in their simplicity, low computational demand, and suitability for low cost applications, particularly in resource constrained embedded devices. In contrast, the TMEAD-BiLSTM model offers high accuracy but requires high performance computing support, making it suitable for applications with high accuracy requirements and ample computational resources. Considering the potential of drones in monitoring alfalfa growth, we plan to lightweighten the model in future work and deploy it on drones to evaluate the method in real-world environments.

## Data Availability

The original contributions presented in the study are included in the article/supplementary material. Further inquiries can be directed to the corresponding author.
